# Combination of left ventricular reverse remodeling and brain natriuretic peptide level at one year after cardiac resynchronization therapy predicts long-term clinical outcome

**DOI:** 10.1371/journal.pone.0219966

**Published:** 2019-07-17

**Authors:** Tomas Roubicek, Jan Stros, Pavel Kucera, Pavel Nedbal, Jan Cerny, Rostislav Polasek, Dan Wichterle

**Affiliations:** 1 Department of Cardiology, Regional Hospital Liberec, Liberec, Czech Republic; 2 Department of Cardiology, Institute for Clinical and Experimental Medicine, Prague, Czech Republic; 3 Faculty of Health Studies, Technical University of Liberec, Liberec, Czech Republic; Scuola Superiore Sant’Anna, ITALY

## Abstract

**Introduction:**

The aim of this study was to investigate the predictors of long-term clinical outcome of heart failure (HF) patients who survived first year after initiation of cardiac resynchronization therapy (CRT).

**Methods:**

This was a single-center observational cohort study of CRT patients implanted because of symptomatic HF with reduced ejection fraction between 2005 and 2013. Left ventricle (LV) diameters and ejection fraction, New York Heart Association (NYHA) class, and level of N-terminal fragment of pro-brain natriuretic peptide (NT-proBNP) were assessed at baseline and 12 months after CRT implantation. Their predictive power for long-term HF hospitalization and mortality, and cardiac and all-cause mortality was investigated.

**Results:**

A total of 315 patients with left bundle branch block or intraventricular conduction delay who survived >1 year after CRT implantation were analyzed in the current study. During a follow-up period of 4.8±2.1 years from CRT implantation, 35.2% patients died from cardiac (19.3%) or non-cardiac (15.9%) causes. Post-CRT LV ejection fraction and LV end-systolic diameter (either 12-month value or the change from baseline) were equally predictive for clinical events. For NT-proBNP, however, the 12-month level was a stronger predictor than the change from baseline. Both reverse LV remodeling and 12-month level of NT-proBNP were independent and comparable predictors of CRT-related clinical outcome, while NT-proBNP response had the strongest association with all-cause mortality. When post-CRT relative change of LV end-systolic diameter and 12-month level of NT-proBNP (dichotomized at -12.3% and 1230 ng/L, respectively) were combined, subgroups of very-high and very-low risk patients were identified.

**Conclusion:**

The level of NT-proBNP and reverse LV remodeling at one year after CRT are independent and complementary predictors of future clinical events. Their combination may help to improve the risk stratification of CRT patients.

## Introduction

Cardiac resynchronization therapy (CRT) has become an established and important treatment for chronic heart failure (HF) patients with left ventricular (LV) systolic dysfunction and left bundle branch block (LBBB) [1–3]. However, approximately 30% of patients fail to respond to CRT [4]. There is a great interest in the early identification not only of determinants of CRT response but also predictors of future clinical events.

In our previous study [5], we showed that electrical LV lead position at implant assessed by Q-LV ratio (electrical delay from the beginning of the QRS complex to the local LV electrogram/QRS duration) was found to be a significant predictor of mortality in CRT patients. In the same population with prolonged follow up, we investigated the long-term prognostic value of short-term (1-year) CRT response on top of baseline clinical characteristics. Specifically, we focused on endsystolic LV diameter, NYHA class and NT-proBNP, either in absolute 12-month values as well as their relative change compared to baseline. Heart failure hospitalizations, heart failure death, cardiac death and all-cause mortality were pre-specified study endpoints.

## Methods

### Patient cohort

Similarly, as in our previous study [5], we retrospectively analyzed data from a prospective database of patients in whom de novo biventricular pacemaker (CRT-P) or defibrillator (CRT-D) was implanted at the Regional Hospital Liberec, Czech Republic between June 2005 and December 2013. All patients signed an informed consent with the procedure. CRT was indicated according to current guidelines of the European Society of Cardiology: symptomatic chronic HF despite optimal medical therapy, LV ejection fraction (LVEF) ≤35% and QRS duration (QRSd) ≥120ms [6]. Only patients with LBBB or intraventricular conduction delay (IVCD) defined according to the Strauss criteria [7] were included. Patients who died prior to 12-month visit were excluded. The study was performed in accordance with the Declaration of Helsinki guidelines and the analysis was approved by the local Ethics Committee.

The right ventricular lead was commonly placed in the midseptum region. The LV lead was inserted transvenously with a preference for lateral followed by posterolateral cardiac veins. Whenever possible, attempts were made to maximize the left ventricular lead electrical delay (Q-LV) at implant. Empirical atrioventricular delay of 120 ms and zero V-V delay were programmed at implant and were not routinely optimized. When no clinical improvement was observed in follow-up visits, patients underwent at least one session of echocardiographic CRT optimization.

### Follow-up

All patients were seen in the local outpatient department every 6 months. In the visits, the results of clinical examination, standard ECG, CRT device settings, medical treatment, and echocardiographic findings were recorded. Clinical outcome data were collected from other relevant medical records, by contacting primary care physicians and the National Health Care mortality registry. When the proportion of ventricular pacing in patients with atrial fibrillation was <90% despite medical therapy, atrioventricular junction ablation was performed. The follow up was completed in July 2016.

### Laboratory assay

Blood samples in tubes containing serum-separating gel for N-terminal fragment of pro-brain natriuretic peptide (NT-proBNP) analysis were collected at baseline and at 12-months follow-up visit. Samples were taken in the morning (8 a.m.) before CRT implantation and in ambulatory setting (12-months visit). Samples at room temperature (20–25 °C) were immediately (< 1 hour) transported for the analysis. Serum / plasma NT-proBNP was measured on a Cobas e411 analyzer (Roche Diagnostics) using the Elecsys proBNP II immunoassay (Roche Diagnostics). The analytical performance of NT-proBNP assay in the reference laboratory of the study was assessed at the level of 140 ng/L (intermediate precision: 2.52%, bias: 2.73%, and combined uncertainty: 5.03%) and at the level of 4810 ng/L (intermediate precision: 1.82%, bias: 2.85%, and combined uncertainty: 3.65%).

### Study endpoints

Four study endpoints were defined for the follow up after first year: HF hospitalization, HF mortality, cardiac mortality, and all-cause mortality. HF hospitalization was defined as a hospital admission with overnight stay because of signs or symptoms of HF, with subsequent improvement with medical therapy. All HF hospitalizations within the first year after CRT implantation were disregarded. The cause of death was assessed by the consensus of two physicians. This was done by careful review of clinical, death and autopsy reports, and CRT device memory when available. Heart failure mortality was defined as death following a progressive deterioration of heart failure symptoms over a period of weeks or months, and which did not fulfil criteria for sudden cardiac death. Cardiac mortality was defined as any death due to cardiac causes, including sudden cardiac death, heart failure death and death due to myocardial infarction. Any sudden death of uncertain cause was considered sudden cardiac death.

### Statistical analysis

Continuous variables were expressed as a mean ± standard deviation and compared by two-tailed t-test for independent samples or Mann-Whitney U test for non-normally distributed data. Categorical variables were expressed as percentages and compared by Chi-square test. Associations of clinical characteristics (including their change during the first year of follow up) with all study endpoints were investigated by Cox proportional-hazards regression analysis with individual factors as continuous variables whenever possible. The NT-proBNP data were log-transformed prior to this analysis. All factors that were univariably associated (P<0.20) with at least one study endpoint were entered into the multivariable Cox regression models and investigated by stepwise-forward method. Predictive power of continuous factors was compared by area under the curve (AUC) by an analysis of receiver-operating characteristics (ROC) curves. Optimum cut-off values were found by the criterion of minimum distance from the [0;1]-point of ROC curve.

In dichotomized population, Kaplan-Meier curves were used to display cumulative event-free survival and the hazard ratios for high-risk subgroups were assessed by Cox proportional-hazards regression analysis. Similarly, combinations of risk factors were investigated. The index of net reclassification improvement was used to quantify the prediction value added by newly proposed risk factors [8]. Starting point for all survival analyses was set at 12-month post-CRT visit. A P-value ≤0.05 was considered significant. Statistical analyses were performed using the STATISTICA vers. 12 software (Statsoft, Inc.) and „easyROC” web-tool for ROC curve analysis (ver. 1.3) [9].

## Results

A total of 328 consecutive patients with LBBB or IVCD with first-time implantation of CRT pacemaker (n = 79) or defibrillator (n = 249) were included. Thirteen patients died within the first year. These patients did not differ from the rest of the study population except for a higher baseline New York Heart Association (NYHA) class (3.5±0.5 vs. 3.1±0.5, P = 0.007) and higher baseline NT-proBNP levels (9609±7744 vs. 3033±4289 ng/L, P˂0.0001).

A total of 315 CRT patients who survived >1 year after CRT implantation were analyzed in the current study. Among those patients, 22 heart failure hospitalizations that occurred before the 12-month visit were disregarded. Patient baseline and 12-month characteristics are shown in Table 1.

**Table 1:** Baseline and 12-month characteristics of study population (N = 315).

Variable	Baseline	12 months
**Males (%)**	76.2	-
**Age (years)**	67±9	-
**Ischemic cardiomyopathy (%)**	56.5	-
**Left bundle branch block (%)**	81.3	-
**Atrial fibrillation (%)**	15.2	-
**Left atrium diameter (mm)**	48.7±6.0	-
**Creatinine (μmol/L)**	103±40	-
**Implantable cardioverter-defibrillator (%)**	75.9	-
**Q-LV (ms)**	122±30	-
**Q-LV ratio**	0.76±0.14	-
**Biventricular capture (%)**		97.4±4.0
**QRS duration (ms)**	161±20	138±19
**NYHA class**	3.1±0.5	2.1±0.7
**LV ejection fraction (%)**	26.2±5.5	38.8±13.8
**LV end-diastolic diameter (mm)**	65.7±7.1	60.7±9.0
**LV end-systolic diameter (mm)**	56.2±8.0	48.4±12.0
**Mitral regurgitation (grade)**	1.7±1.0	1.3±0.7
**NT-proBNP (ng/L)**	1672 (871–3603)	952 (423–2519)

The values are percentage, mean ± standard deviation or median (interquartile range). Atrial fibrillation category includes persistent or permanent form of arrhythmia. Left bundle branch block was defined according to criteria by Strauss. Abbreviations: NT-proBNP = N-terminal fragment of pro-brain natriuretic peptide, NYHA = New York Heart Association; LV = left ventricle; Q-LV = left ventricular lead local electrogram delay from the QRS onset; Q-LV ratio = Q-LV / QRS duration.

Patients were treated with beta-blockers (96%), angiotensin-converting enzyme inhibitors or angiotensin receptor blockers (99%), loop diuretics (91%), and mineralocorticoid-receptor antagonists (89%). Quadripolar LV lead was used only in 11 patients. Only one patient was upgraded from CRT-P to CRT-D due to occurrence of ventricular tachycardia. Ten patients with CRT-D underwent the radiofrequency ablation for ventricular tachycardia during the follow up.

During the mean follow-up period of 4.8±2.1 years (median: 4.6 years) from CRT implantation, 82 patients (26%) were hospitalized for heart failure and 111 (35.2%) patients died of cardiac (n = 61, 19.3%) or non-cardiac (n = 50, 15.9%) causes. Cardiac deaths were due to heart failure (14.2%), sudden cardiac death (4.1%) and other cardiac reasons (1%). The risk of sudden death was higher in patients with CRT-P (7/76 = 9.2%) compared to CRT-D (6/239 = 2.5%), P = 0.02.

Differences between groups of patients defined by clinical outcome after the 12-months visit (HF hospitalization and death, cardiac and all-cause death) can be found in S1 and S2 Tables, and univariate associations between individual factors and clinical events are shown in Tables 2 and 3.

**Table 2:** Univariate association between individual factors and clinical events (hospitalization and death due to heart failure).

	Heart failure hospitalization N = 82			Heart failure death N = 45		
	HR	95% CI	P-value	HR	95% CI	P-value
**Male gender (1/0)**	1.8	1.01–3.3	0.046	2.2	0.95–5.3	0.06
**Age (years)**	1.6	0.99–2.4	0.053	1.02	0.99–1.1	0.21
**Ischemic cardiomyopathy (1/0)**	1.6	0.99–2.4	0.053	2.9	1.5–5.7	0.002
**Non-left bundle branch block (1/0)**	1.6	0.96–2.7	0.07	1.6	0.83–3.2	0.15
**Atrial fibrillation (1/0)**	0.76	0.39–1.5	0.41	0.89	0.37–2.1	0.78
**Left atrium diameter (mm)**	1.1	1.04–1.1	0.00006	1.1	1.1–1.2	0.00002
**Creatinine (μmol/L)**	1.01	1.00–1.01	0.003	1.00	1.00–1.01	0.42
**Biventricular pacemaker only (1/0)**	1.2	0.73–1.9	0.53	0.85	0.43–1.7	0.64
**Q-LV (ms)**	0.99	0.99–1.00	0.07	0.99	0.98–1.00	0.02
**Q-LV ratio**	0.15	0.03–0.69	0.01	0.06	0.01–0.34	0.002
**Biventricular capture (%)**	0.95	0.91–0.99	0.03	0.92	0.87–0.97	0.001
**QRS duration—baseline (ms)**	1.00	0.99–1.01	0.92	1.00	0.99–1.02	0.78
**QRS duration—post-CRT (ms)**	1.01	1.00–1.02	0.21	1.00	0.99–1.02	0.75
**QRS duration—relative change (%)**	1.01	0.99–1.03	0.30	1.00	0.98–1.02	0.93
**NYHA Class—baseline (2/3/4)**	1.8	1.2–2.8	0.005	1.5	0.85–2.6	0.16
**NYHA Class—month 12 (2/3/4)**	2.0	1.5–2.7	<0.00001	2.1	1.4–3.1	0.0001
**NYHA Class—change**	1.4	1.03–1.9	0.03	1.7	1.1–2.6	0.009
**LV ejection fraction—baseline (%)**	0.95	0.91–0.99	0.01	0.95	0.89–1.00	0.047
**LV ejection fraction—month 12 (%)**	0.95	0.93–0.97	<0.00001	0.93	0.90–0.95	<0.00001
**LV ejection fraction—relative change (%)**	0.99	0.99–0.99	0.00004	0.98	0.97–0.99	<0.00001
**LV end-diastolic diameter—baseline (mm)**	1.02	0.99–1.1	0.29	1.02	0.98–1.1	0.32
**LV end-diastolic diameter—month 12 (mm)**	1.1	1.03–1.1	<0.00001	1.1	1.04–1.1	<0.00001
**LV end-diastolic diameter—relative change (%)**	1.1	1.04–1.1	<0.00001	1.1	1.1–1.1	<0.00001
**LV end-systolic diameter—baseline (mm)**	1.02	0.99–1.1	0.15	1.03	0.99–1.1	0.20
**LV end-systolic diameter—month 12 (mm)**	1.05	1.03–1.1	<0.00001	1.1	1.04–1.1	<0.00001
**LV end-systolic diameter—relative change (%)**	1.05	1.03–1.1	<0.00001	1.1	1.05–1.1	<0.00001
**Mitral regurgitation—baseline (1/2/3/4)**	1.1	0.86–1.3	0.54	1.02	0.76–1.4	0.91
**Mitral regurgitation—month 12 (1/2/3/4)**	1.8	1.5–2.3	<0.00001	1.8	1.4–2.3	0.00003
**Mitral regurgitation—change**	1.6	1.2–2.1	0.002	1.8	1.2–2.6	0.004
**NT-proBNP—baseline (log ng/L)**	2.2	1.3–3.5	0.002	3.1	1.6–6.1	0.0009
**NT-proBNP—month 12 (log ng/L)**	5.2	3.2–8.5	<0.00001	5.8	3.1–10.8	<0.00001
**NT-proBNP—change**	3.5	2.0–6.0	<0.00001	3.0	1.5–6.2	0.003

CI = confidence interval; HR = hazard ratio; log = decadic logarithm; for other abbreviations see the Table 1.

**Table 3:** Univariate association between individual factors and clinical events (cardiac and all-cause mortality).

	Cardiac death N = 61			All-cause death N = 111		
	HR	95% CI	P-value	HR	95% CI	P-value
**Male gender (1/0)**	2.0	0.98–4.0	0.06	1.5	0.91–2.4	0.11
**Age (years)**	1.03	1.00–1.1	0.04	1.04	1.02–1.1	0.0004
**Ischemic cardiomyopathy (1/0)**	2.6	1.5–4.6	0.001	2.3	1.5–3.5	0.00008
**Non-left bundle branch block (1/0)**	1.4	0.75–2.5	0.31	1.3	0.83–2.1	0.25
**Atrial fibrillation (1/0)**	0.87	0.41–1.8	0.71	1.2	0.73–2.0	0.49
**Left atrium diameter (mm)**	1.1	1.1–1.2	<0.00001	1.1	1.04–1.1	0.00001
**Creatinine (μmol/L)**	1.01	1.00–1.01	0.001	1.00	1.00–1.01	0.002
**Biventricular pacemaker only (1/0)**	1.3	0.76–2.2	0.34	1.2	0.78–1.8	0.44
**Q-LV (ms)**	0.99	0.99–1.00	0.17	1.00	0.99–1.00	0.50
**Q-LV ratio**	0.15	0.03–0.76	0.02	0.33	0.09–1.1	0.08
**Biventricular capture (%)**	0.91	0.87–0.95	0.00003	0.93	0.90–0.97	0.0001
**QRS duration—baseline (ms)**	1.00	0.99–1.02	0.53	1.01	1.00–1.02	0.22
**QRS duration—post-CRT (ms)**	1.00	0.99–1.02	0.79	1.01	1.00–1.02	0.20
**QRS duration—relative change (%)**	1.00	0.98–1.02	0.73	1.00	0.99–1.02	0.98
**NYHA Class—baseline (2/3/4)**	1.6	0.98–2.6	0.06	1.1	0.80–1.7	0.46
**NYHA Class—month 12 (2/3/4)**	1.9	1.3–2.6	0.0002	1.7	1.3–2.1	0.0001
**NYHA Class—change**	1.5	1.02–2.1	0.04	1.5	1.2–2.0	0.002
**LV ejection fraction—baseline (%)**	0.98	0.93–1.02	0.33	1.00	0.96–1.03	0.94
**LV ejection fraction—month 12 (%)**	0.94	0.92–0.96	<0.00001	0.97	0.95–0.98	<0.00001
**LV ejection fraction—relative change (%)**	0.98	0.98–0.99	<0.00001	0.99	0.99–0.99	<0.00001
**LV end-diastolic diameter—baseline (mm)**	1.02	0.98–1.1	0.41	1.01	0.98–1.04	0.42
**LV end-diastolic diameter—month 12 (mm)**	1.1	1.04–1.1	<0.00001	1.05	1.03–1.1	0.00001
**LV end-diastolic diameter—relative change (%)**	1.1	1.1–1.1	<0.00001	1.1	1.04–1.1	<0.00001
**LV end-systolic diameter—baseline (mm)**	1.01	0.98–1.04	0.51	1.01	0.99–1.03	0.44
**LV end-systolic diameter—month 12 (mm)**	1.1	1.03–1.1	<0.00001	1.04	1.02–1.1	<0.00001
**LV end-systolic diameter—relative change (%)**	1.1	1.04–1.1	<0.00001	1.04	1.02–1.1	<0.00001
**Mitral regurgitation—baseline (1/2/3/4)**	1.03	0.81–1.3	0.81	1.1	0.89–1.3	0.46
**Mitral regurgitation—month 12 (1/2/3/4)**	1.6	1.2–2.1	0.0003	1.4	1.2–1.8	0.0004
**Mitral regurgitation—change**	1.4	1.05–2.0	0.03	1.2	0.96–1.5	0.10
**NT-proBNP—baseline (log ng/L)**	3.4	1.9–6.1	0.00003	2.6	1.7–4.1	<0.00001
**NT-proBNP—month 12 (log ng/L)**	6.6	3.9–11.2	<0.00001	4.7	3.2–7.0	<0.00001
**NT-proBNP—change**	3.2	1.7–6.0	0.0002	2.7	1.7–4.3	0.00002

CI = confidence interval; HR = hazard ratio; log = decadic logarithm; for other abbreviations see the Table 1.

According to the results of multivariate analysis, which are shown in Table 4, the 12-month level of NT-proBNP was an independent predictor of clinical outcome that was consistently associated with all study endpoints. On the other hand, various indices of LV morphology / function (expressed as either the first-year change or final 12-month value) mutually competed and, therefore, did not consistently demonstrate independent association with clinical outcome.

**Table 4:** Multivariate predictors of clinical events.

	Heart failure hospitalization N = 82			Heart failure death N = 45			Cardiac death N = 61			All-cause death N = 111		
	Chi-square = 74.6			Chi-square = 64.3			Chi-square = 80.0			Chi-square = 92.2		
	R = 0.37, P <0.0001			R = 0.36, P <0.0001			R = 0.42, P <0.0001			R = 0.47, P <0.0001		
	HR	95% CI	P-value	HR	95% CI	P-value	HR	95% CI	P-value	HR	95% CI	P-value
**Age (years)**										1.03	1.01–1.1	0.02
**Ischemic cardiomyopathy (1/0)**				2.5	1.2–4.9	0.01	2.3	1.3–4.1	0.005	2.0	1.3–3.0	0.002
**Left atrium diameter (mm)**				1.1	1.01–1.1	0.01	1.1	1.01–1.1	0.02	1.04	1.00–1.1	0.04
**Biventricular capture (%)**										0.96	0.93–1.00	0.045
**NYHA Class—baseline (2/3/4)**	1.8	1.2–2.7	0.008									
**LV ejection fraction—month 12 (%)**	0.97	0.95–0.99	0.002									
**LV ejection fraction—relative change (%)**										1.00	0.99–1.00	0.03
**LV end-systolic diameter—relative change (%)**				1.05	1.02–1.1	0.0002	1.04	1.02–1.1	0.0005			
**Mitral regurgitation—change**	1.7	1.3–2.2	0.0001									
**NT-proBNP—month 12 (log ng/L)**	3.6	2.1–6.2	<0.00001	3.6	1.7–7.7	0.0009	4.6	2.4–8.6	<0.00001	3.3	2.1–5.3	<0.00001

Results are provided only for factors that were significantly associated with at least one clinical endpoint. HR = hazard ratio; CI = confidence interval; log = decadic logarithm; for other abbreviations see the Table 1.

**Fig 1:**
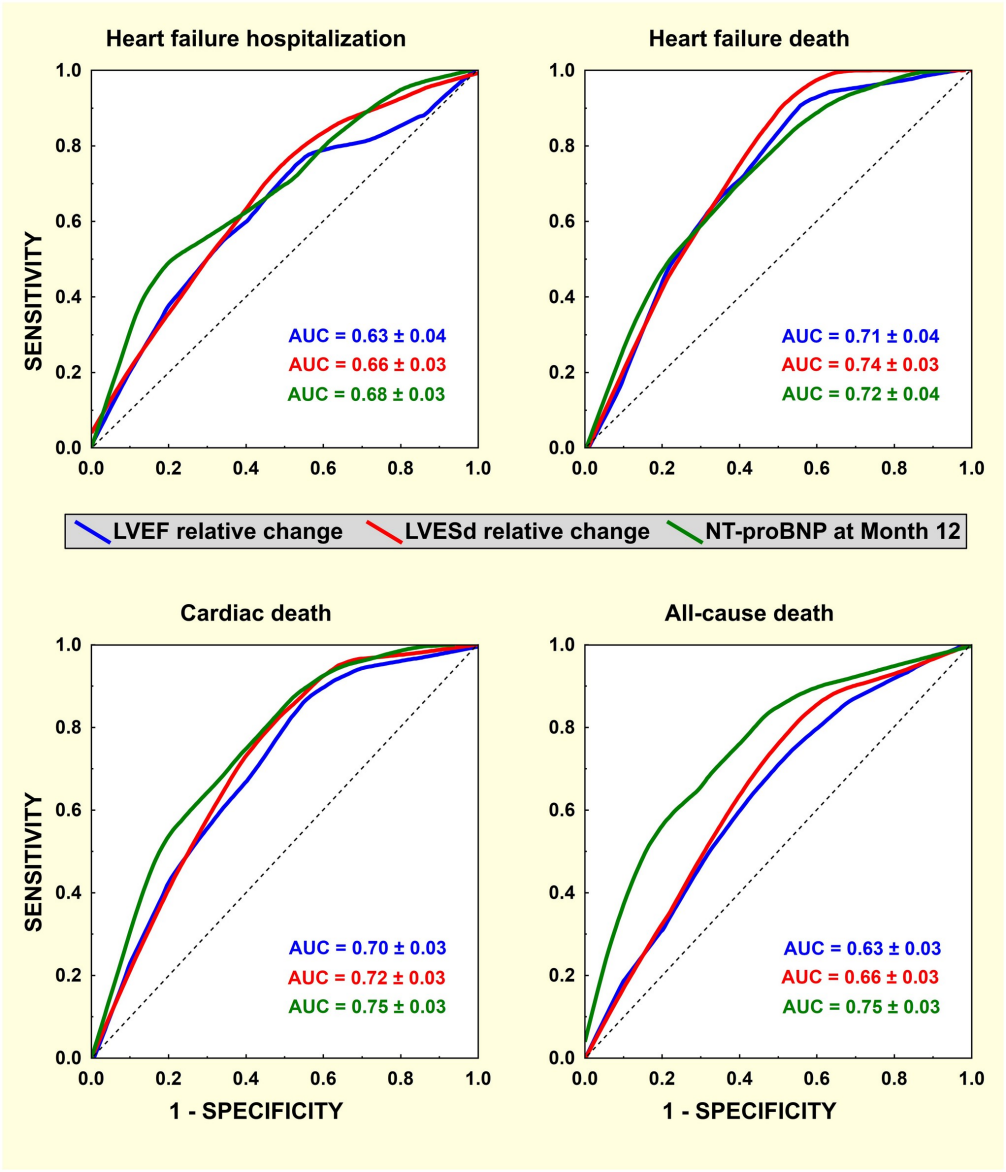
Receiver-operating characteristics for 12-month response to CRT and clinical events. Receiver-operating curves for post-CRT relative change in LV ejection fraction (LVEF, blue), end-systolic diameter (LVESd, red) and 12-month NT-proBNP level (green) and subsequent clinical events (heart failure hospitalization / death and cardiac/ all-cause death). The curves were obtained by locally weighted scatterplot smoothing. Areas under the curve with standard error are shown inside the graphs.

Therefore, we selected NT-proBNP, LV end-systolic diameter (LVESd) and ejection fraction (LVEF) for a direct comparison of their predictive power by the analysis of ROC curves for all clinical endpoints. First, we compared the predictive power of their 12-month values versus the first-year change (Table 5). Except for heart failure hospitalization, survival was predicted significantly better by the 12-month level compared to the relative change in NT-proBNP. On the contrary, the final value and change during the first year were comparably predictive in the case of LVESd and LVEF. Second, we compared NT-proBNP at the 12-month visit and the relative change of LVESd and LVEF. In this more extensive analysis (cross-tabulated results are not shown), the predictive characteristics of all investigated indices were comparable with the exception of NT-proBNP at 12-month visit, which significantly outperformed the relative change of both LVESd and LVEF, but this was only valid for all-cause mortality (Fig 1).

**Table 5:** Receiver-operating characteristics: Comparison of areas under the curve.

	LV ejection fraction			LV end-systolic diameter			NT-proBNP		
	12-month value	relative change	P	12-month value	relative change	P	12-month value	relative change	P
**Heart failure hospitalization**	0.68±0.05	0.63±0.05	0.34	0.65±0.01	0.66±0.05	0.81	0.68±0.05	0.63±0.05	0.27
**Heart failure death**	0.75±0.03	0.71±0.05	0.54	0.72±0.03	0.74±0.05	0.57	0.72±0.12	0.59±0.06	0.03
**Cardiac death**	0.71±0.01	0.70±0.05	0.88	0.68±0.03	0.72±0.05	0.48	0.75±0.14	0.61±0.05	0.006
**All-cause death**	0.65±0.01	0.63±0.05	0.79	0.65±0.01	0.66±0.04	0.84	0.75±0.12	0.63±0.04	0.005

The values are areas under the curve ± standard error. For abbreviations see the Table 1.

Table 6 shows optimal cut-off values together with predictive characteristics derived from ROC analysis. Cut-offs were rather uniform for individual clinical endpoints, so that the dichotomies that were obtained for cardiac death were subsequently used also for other clinical endpoints.

**Table 6:** Receiver-operating characteristics: Optimum cut-off values.

	LV ejection fraction (relative change)			LV end-systolic diameter (relative change)			NT-proBNP (at Month 12)		
	Cut-off value	Sensitivity	Specificity	Cut-off value	Sensitivity	Specificity	Cut-off value	Sensitivity	Specificity
**Heart failure hospitalization**	+33.3%	60%	63%	-12.7%	68%	58%	1170 ng/L	61%	65%
**Heart failure death**	+33.3%	73%	62%	-12.3%	84%	58%	1184 ng/L	69%	63%
**Cardiac death**	+34.5%	69%	63%	-12.3%	79%	59%	1230 ng/L	69%	67%
**All-cause death**	+34.8%	59%	64%	-13.2%	69%	58%	1289 ng/L	63%	75%

Optimum cut-off values were defined by the point with the shortest distance from the [0,1]-point of the receiver-operating graph. For abbreviations see the Table 1.

**Fig 2:**
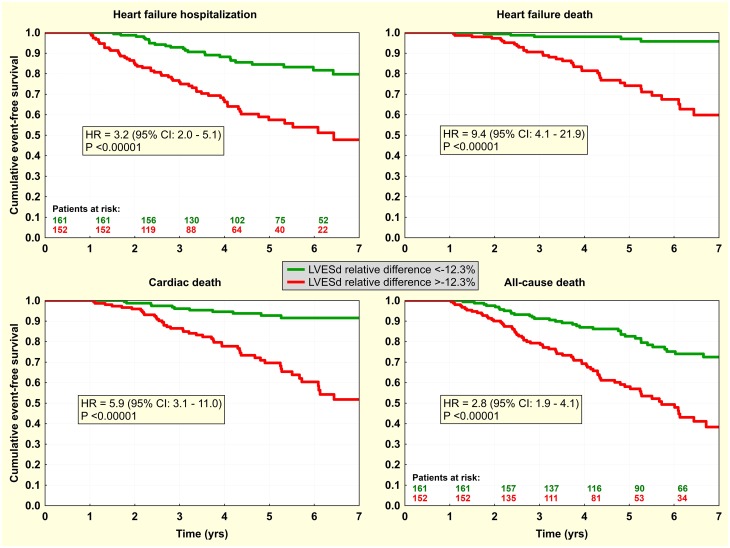
Event-free survival according to post-CRT reverse left ventricular remodeling. Kaplan-Meier curves for the first heart failure hospitalization / death and cardiac / all-cause death according to relative change in left ventricle end-systolic diameter (LVESd) with dichotomy of -12.3% relative change.

**Fig 3:**
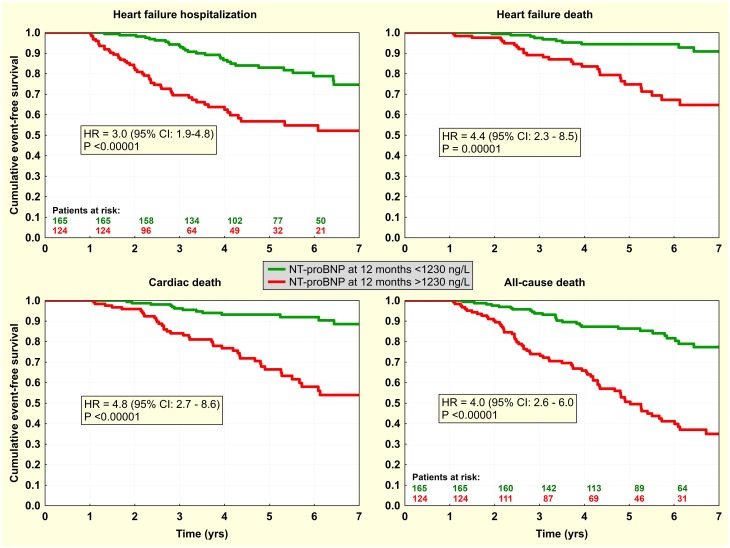
Event-free survival according to post-CRT level of NT-proBNP. Kaplan-Meier curves for the first heart failure hospitalization / death and cardiac / all-cause death according to 12-month NT-proBNP level with dichotomy of 1230 ng/L.

**Fig 4:**
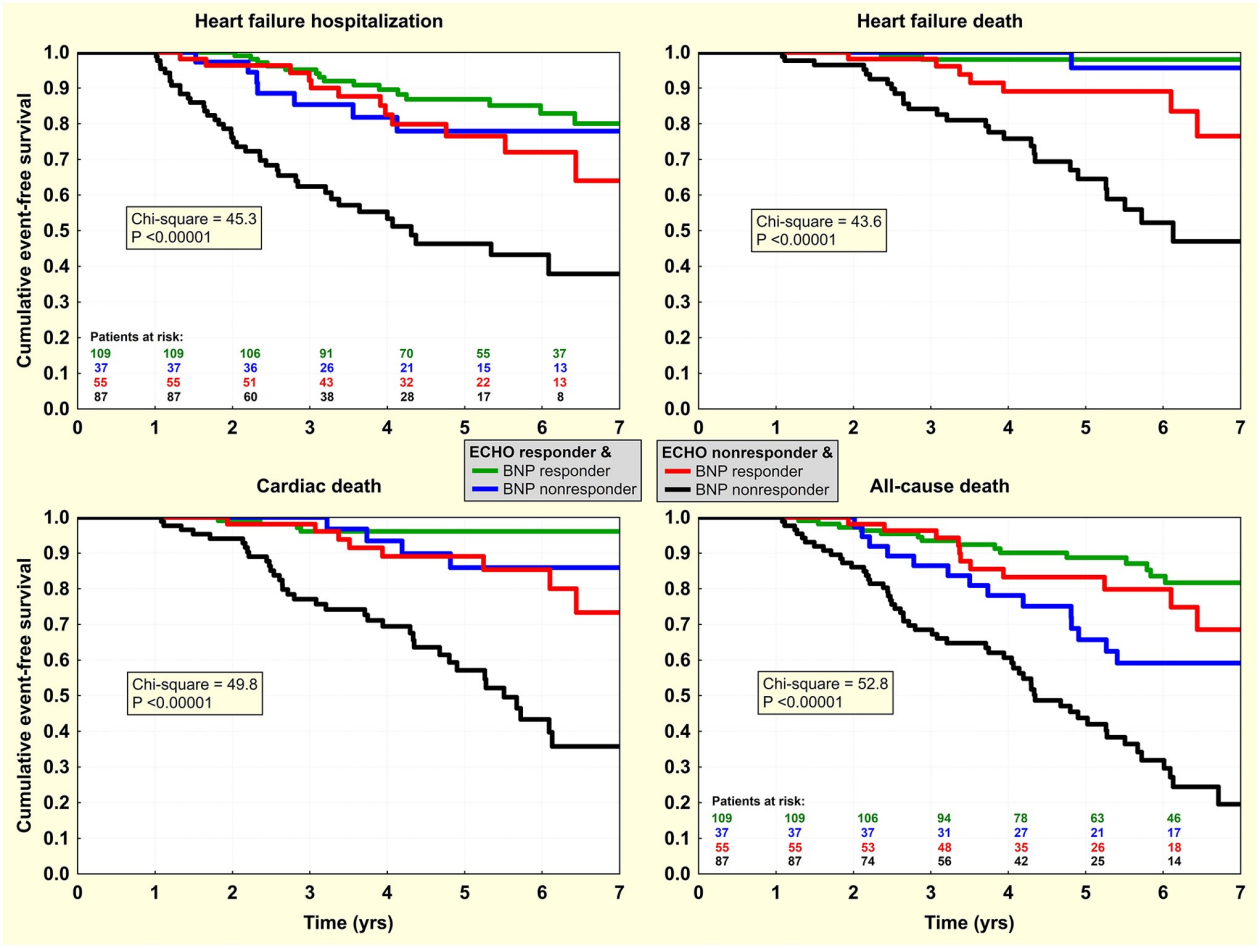
Event-free survival in 4 categories of post-CRT response. Kaplan-Meier curves for the first heart failure hospitalization / death and cardiac / all-cause death according to „ECHO response”(LVESd relative change <-12.3%) and „BNP response”(NT-proBNP <1230 ng/L at Month 12 after CRT).

Finally, dichotomized predictors were investigated by Kaplan-Meier analysis. Knowing the interchangeability of markers of reverse LV remodeling, this was done only for LVESd with the dichotomy of -12.3% for its relative change (Fig 2) and for 12-month level of NT-proBNP with a dichotomy of 1230 ng/L (Fig 3). In multivariate analysis, both factors were statistically independent, and this was preserved even after adjustment for other clinical characteristics, either continuous or dichotomized. Combination of both factors identified subgroups of very-high and very-low risk patients (Fig 4). Inclusion of NT-proBNP to simple LVESd-based risk stratification (when only patients with simultaneous change in LVESd >-12.3% and NT-proBNP >1230 ng/L were considered high-risk) resulted in net reclassification improvement of 10.8%, 14.2%, 13.5%, and 11.5% for HF hospitalization, HF death, cardiac death, and all-cause death, respectively.

## Discussion

This single-center study with a long follow up suggested that the level of NT-proBNP and indices of reverse LV remodeling at one year after CRT implantation are replaceable predictors of future clinical events. Their predictive power was independent, and their combination improved the risk stratification of CRT patients. Another important finding was that the 12-month level of NT-proBNP appeared to be a significantly stronger outcome predictor than its change post-implantation. Conversely, the predictive power of echocardiographic indices (LVESd and LVEF) was comparable for both absolute 12-month values and relative change.

It has been previously demonstrated that uneventful survival of CRT patients is tightly associated with echocardiographic response, and responders have a better prognosis overall [10–17]. It has also been shown that there is considerable disagreement between clinical (NYHA-based) and echocardiographic CRT response [18,19] suggesting that their combination could potentially result in a stronger composite risk predictor. On the other hand, only a single study confirmed the independent predictive power of post-CRT improvement in NYHA class [15], while other studies on the predictive value of clinical CRT response were either negative [10,12] or confirmed that this association was not significant in multivariate analysis when the echocardiographic CRT response was considered [14]. Similarly, our study did not confirm the utility of NYHA class change when adjusted for other predictors.

Having in mind the subjective nature of the assessment of clinical CRT response (without systematic use of 6 minute walking test or a quality life questionnaire in daily practice), natriuretic peptide levels have been suggested for the monitoring of CRT patients in numerous small studies with short follow up [20–27]. Arrigo et al. [28] found that low circulating mid-regional-pro-atrial natriuretic peptide at the time of device implantation is associated with CRT response and more favorable outcome. An analysis of the CARE-HF study [29] showed that CRT exerts an early and sustained reduction in NT-proBNP [30] and that patients with more severe mitral regurgitation or persistently elevated NT-proBNP despite adequate treatment of heart failure have a higher mortality [31]. In the CRT arm of the MADIT-CRT trial [3], patients in whom 1-year BNP levels were reduced or remained low experienced a significantly lower risk of subsequent HF or death as compared with patients in whom 1-year BNP levels were high [32].

In a study by Hoogslag et al. [33], a left ventricular end-systolic volume (LVESV) and NT-proBNP reduction ≥15% independently predicted the clinical outcome. Bakos et al. [34] proposed a composite score consisting of clinical, echocardiographic (LVESV reduction ≥15%) and humoral response (NT-proBNP reduction ≥25%), which predicted the combined endpoint of mortality and HF events.

In the present study, we did not find any significant value of the clinical response for the prediction of post-CRT risk, whereas the combination of echocardiographic and humoral markers was particularly useful to refine the risk stratification in this context. Indeed, the most divergent survival curves were found for dual responders compared to dual nonresponders. More importantly, our study questioned the utility of relative post-CRT change of individual markers for the prediction of subsequent clinical outcome, suggesting that the absolute 12-month values of individual indices are equally predictive (or even better predictive in the case of NT-proBNP) when compared to the post-CRT change. Such observation appears logically sound, as the post-CRT change is more applicable for the assessment (or comparison) of early treatment effects, while the 12-month state may be more relevant for subsequent clinical outcome than any baseline conditions or CRT-induced improvement. The confirmation of these findings with retrospective analyses of randomized studies could provide valuable insight into this matter.

The incidence of sudden death was low in our study (4.1%) which precluded reliable risk stratification. Therefore, corresponding data were not presented as for other study endpoints. The risk of sudden death was higher in CRT-P recipients. However, CRT-P patients were significantly older, had significantly more comorbidities and significantly higher non-sudden mortality. In multivariate analysis, only elevated NT-proBNP was associated with sudden death while absence of ICD was not significant risk factor.

### Study implications

This study expands on the current knowledge on the impact of early response to CRT implantation on subsequent clinical events. Clinical response was the weakest predictor of long-term outcome and should be used only when other objective measures are not available. The combination of echocardiographic and humoral response improved the identification of patients at risk. Such patients may benefit from escalated HF therapy, CRT optimization or reintervention including LV lead reimplant (either transvenously, endocardially or surgically), or the use of multipoint/multisite pacing. Despite the combination of risk factor, their overall predictive power for clinical events is, however, relatively modest and of limited practical utility.

### Study limitations

Although the data in our CRT database were collected prospectively, the hypotheses were defined post-hoc and data analyzed retrospectively. Therefore, the results should be interpreted with caution. The cut-off values for risk prediction may not be applicable to different patient populations. Furthermore, the usefulness of the different risk predictors may have been overestimated as cut-off values were optimized for our single-center population. The relative low number of events (especially mortality) found in the study could possibly affect the statistical power of regressions analysis including several variables and subgroups of patients. Finally, we used LVESd as an index of LV reverse remodeling instead of LVESV. However, the lower accuracy of LVESd compared to LVESV may be compensated by its higher reproducibility and ease of access.

## Conclusions

Absence of both echocardiographic and humoral response one year after the CRT implant identifies patients at the highest risk of heart failure progression and death. Such patients are good candidates for advanced HF management.

## Supporting information

S1 Table Comparison of baseline and 12-month characteristics in subgroups according to clinical events (hospitalization and death due to heart failure). The values are percentage or mean ± standard deviation. NS = not significant; for other abbreviations see the Table 1.
(DOCX)

S2 Table Comparison of baseline and 12-month characteristics in subgroups according to clinical events (cardiac and all-cause death). The values are percentage or mean ± standard deviation. NS = not significant; for other abbreviations see the Table 1.
(DOCX)
