# Supplementary material for: Combination of left ventricular reverse remodeling and brain natriuretic peptide level at one year after cardiac resynchronization therapy predicts long-term clinical outcome
Source: PLoS One. 2019 Jul 17;14(7):e0219966. doi: 10.1371/journal.pone.0219966 (PMC6636764; doi:10.1371/journal.pone.0219966)
Supplement: S1 Table — The values are percentage or mean ± standard deviation. NS = not significant; for other abbreviations see the Table 1. (DOCX) [file pone.0219966.s001.docx]

**S1 Table. Comparison of baseline and 12-month characteristics in subgroups according to clinical events (hospitalization and death due to heart failure)**

|  | **Heart failure hospitalization** | | | **Heart failure death** | | |
| --- | --- | --- | --- | --- | --- | --- |
|  | **NO** | **YES** | **P-value** | **NO** | **YES** | **P-value** |
|  | **N = 233** | **N = 82** |  | **N = 270** | **N = 45** |  |
| **Male gender** | 73.4% | 84.1% | 0.049 | 74.4% | 86.7% | NS |
| **Age (years)** | 67±9 | 68±10 | NS | 67±9 | 68±10 | NS |
| **Ischemic cardiomyopathy** | 54.1% | 63.4% | NS | 53.3% | 75.6% | 0.005 |
| **Non-left bundle branch block** | 17.2% | 23.2% | NS | 17.8% | 24.4% | NS |
| **Atrial fibrillation** | 16.3% | 12.2% | NS | 15.6% | 13.3% | NS |
| **Left atrium diameter (mm)** | 48±6 | 51±6 | 0.0009 | 48±6 | 52±5 | 0.0003 |
| **Creatinine (µmol/L)** | 100±31 | 111±58 | 0.03 | 102±40 | 106±41 | NS |
| **Biventricular pacemaker only** | 21.9% | 30.5% | NS | 23.7% | 26.7% | NS |
| **Q-LV (ms)** | 124±29 | 117±32 | 0.06 | 124±30 | 111±29 | 0.009 |
| **Q-LV ratio** | 0.77±0.14 | 0.72±0.16 | 0.02 | 0.77±0.14 | 0.69±0.13 | 0.0007 |
| **Biventricular capture (%)** | 98±4 | 97±4 | NS | 98±4 | 96±5 | 0.03 |
| **QRS duration - baseline (ms)** | 160±20 | 161±22 | NS | 161±20 | 160±22 | NS |
| **QRS duration - post-CRT (ms)** | 137±19 | 140±19 | NS | 137±19 | 139±18 | NS |
| **QRS duration - relative change (%)** | -14±14 | -12±12 | NS | -13±13 | -13±11 | NS |
| **NYHA Class - baseline (2/3/4)** | 3.0±0.5 | 3.2±0.5 | 0.002 | 3.0±0.5 | 3.2±0.5 | 0.06 |
| **NYHA Class - month 12 (2/3/4)** | 2.1±0.6 | 2.3±0.7 | 0.0007 | 2.1±0.6 | 2.4±0.8 | 0.001 |
| **NYHA Class – change** | -1.0±0.7 | -0.9±0.8 | NS | -1.0±0.7 | -0.8±0.7 | NS |
| **LV ejection fraction - baseline (%)** | 27±5 | 25±6 | 0.001 | 27±5 | 24±5 | 0.006 |
| **LV ejection fraction - month 12 (%)** | 41±14 | 33±13 | <0.00001 | 40±14 | 29±9 | <0.00001 |
| **LV ejection fraction - relative change (%)** | 56±51 | 35±51 | 0.002 | 56±52 | 19±33 | <0.00001 |
| **LV enddiastolic diameter - baseline (mm)** | 65±7 | 67±8 | NS | 66±7 | 67±6 | NS |
| **LV enddiastolic diameter - month 12 (mm)** | 60±9 | 64±9 | 0.0001 | 60±9 | 66±8 | 0.00003 |
| **LV enddiastolic diameter - relative change (%)** | -9±9 | -4±9 | 0.00002 | -9±9 | -2±6 | <0.00001 |
| **LV endsystolic diameter - baseline (mm)** | 56±8 | 57±9 | NS | 56±8 | 58±7 | NS |
| **LV endsystolic diameter - month 12 (mm)** | 47±12 | 53±12 | 0.00003 | 47±12 | 56±9 | <0.00001 |
| **LV endsystolic diameter - relative change (%)** | -17±15 | -8±15 | <0.00001 | -16±16 | -3±9 | <0.00001 |
| **Mitral regurgitation - baseline (1/2/3/4)** | 1.7±1.0 | 1.8±1.0 | NS | 1.7±1.0 | 1.8±1.0 | NS |
| **Mitral regurgitation - month 12 (1/2/3/4)** | 1.2±0.5 | 1.6±1.0 | <0.00001 | 1.2±0.6 | 1.7±1.0 | 0.00007 |
| **Mitral regurgitation - change** | -0.5±0.9 | -0.2±0.9 | 0.02 | -0.5±0.9 | -0.2±1.1 | 0.03 |
| **NT-proBNP - baseline (ng/L)** | 2705±3809 | 3977±5356 | 0.005 | 2777±4019 | 4642±5492 | 0.001 |
| **NT-proBNP - month 12 (ng/L)** | 1471±2005 | 3211±4508 | <0.00001 | 1697±2738 | 3409±3937 | <0.00001 |
| **NT-proBNP - relative change (%)** | -18±102 | 9±104 | 0.0005 | -16±97 | 18±134 | 0.03 |

The values are percentage or mean ± standard deviation.

NS = not significant; for other abbreviations see the Table 1.
