# Supplementary material for: Combination of left ventricular reverse remodeling and brain natriuretic peptide level at one year after cardiac resynchronization therapy predicts long-term clinical outcome
Source: PLoS One. 2019 Jul 17;14(7):e0219966. doi: 10.1371/journal.pone.0219966 (PMC6636764; doi:10.1371/journal.pone.0219966)
Supplement: S2 Table — The values are percentage or mean ± standard deviation. NS = not significant; for other abbreviations see the Table 1. (DOCX) [file pone.0219966.s002.docx]

**S2 Table. Comparison of baseline and 12-month characteristics in subgroups according to clinical events (cardiac and all-cause death)**

|  | **Cardiac death** | | | **All-cause death** | | |
| --- | --- | --- | --- | --- | --- | --- |
|  | **NO** | **YES** | **P-value** | **NO** | **YES** | **P-value** |
|  | **N = 254** | **N = 61** |  | **N = 204** | **N = 111** |  |
| **Male gender** | 74.0% | 85.2% | 0.06 | 73.5% | 81.1% | NS |
| **Age (years)** | 67±9 | 69±9 | NS | 66±9 | 70±8 | 0.0008 |
| **Ischemic cardiomyopathy** | 52.4% | 73.8% | 0.002 | 48.5% | 71.2% | 0.00009 |
| **Non-left bundle branch block** | 18.1% | 21.3% | NS | 17.6% | 20.7% | NS |
| **Atrial fibrillation** | 15.7% | 13.1% | NS | 14.2% | 17.1% | NS |
| **Left atrium diameter (mm)** | 48±6 | 51±5 | 0.00005 | 48±6 | 51±6 | 0.0001 |
| **Creatinine (µmol/L)** | 99±30 | 116±66 | 0.003 | 98±30 | 112±53 | 0.003 |
| **Biventricular pacemaker only** | 21.7% | 34.4% | 0.04 | 19.6% | 32.4% | 0.01 |
| **Q-LV (ms)** | 123±29 | 116±32 | NS | 123±29 | 119±31 | NS |
| **Q-LV ratio** | 0.77±0.14 | 0.71±0.16 | 0.01 | 0.77±0.14 | 0.73±0.15 | 0.02 |
| **Biventricular capture (%)** | 98±3 | 96±6 | 0.002 | 98±3 | 96±5 | 0.003 |
| **QRS duration - baseline (ms)** | 160±20 | 161±21 | NS | 160±20 | 162±22 | NS |
| **QRS duration - post-CRT (ms)** | 137±18 | 139±20 | NS | 136±18 | 140±20 | NS |
| **QRS duration - relative change (%)** | -13±13 | -13±14 | NS | -14±12 | -13±15 | NS |
| **NYHA Class - baseline (2/3/4)** | 3.0±0.5 | 3.2±0.6 | 0.01 | 3.0±0.5 | 3.1±0.6 | NS |
| **NYHA Class - month 12 (2/3/4)** | 2.1±0.6 | 2.4±0.7 | 0.002 | 2.0±0.6 | 2.3±0.7 | 0.001 |
| **NYHA Class – change** | -1.0±0.7 | -0.9±0.7 | NS | -1.0±0.7 | -0.8±0.8 | NS |
| **LV ejection fraction - baseline (%)** | 26±5 | 25±6 | NS | 26±5 | 26±6 | NS |
| **LV ejection fraction - month 12 (%)** | 41±14 | 31±11 | <0.00001 | 41±14 | 34±12 | <0.00001 |
| **LV ejection fraction - relative change (%)** | 57±52 | 23±39 | <0.00001 | 59±53 | 35±44 | 0.00006 |
| **LV enddiastolic diameter - baseline (mm)** | 66±7 | 66±6 | NS | 65±7 | 66±7 | NS |
| **LV enddiastolic diameter - month 12 (mm)** | 60±9 | 65±9 | 0.00003 | 59±9 | 63±9 | 0.00005 |
| **LV enddiastolic diameter - relative change (%)** | -9±9 | -2±8 | <0.00001 | -9±9 | -4±8 | <0.00001 |
| **LV endsystolic diameter - baseline (mm)** | 56±8 | 57±8 | NS | 56±8 | 57±8 | NS |
| **LV endsystolic diameter - month 12 (mm)** | 47±12 | 55±11 | <0.00001 | 46±12 | 52±11 | 0.00004 |
| **LV endsystolic diameter - relative change (%)** | -17±16 | -4±12 | <0.00001 | -17±16 | -9±14 | <0.00001 |
| **Mitral regurgitation - baseline (1/2/3/4)** | 1.7±1.0 | 1.8±1.0 | NS | 1.6±0.9 | 1.8±1.0 | 0.06 |
| **Mitral regurgitation - month 12 (1/2/3/4)** | 1.2±0.6 | 1.5±0.9 | 0.001 | 1.2±0.6 | 1.4±0.9 | 0.001 |
| **Mitral regurgitation - change** | -0.5±0.9 | -0.3±1.0 | NS | -0.5±0.9 | -0.4±1.0 | NS |
| **NT-proBNP - baseline (ng/L)** | 2607±3669 | 4875±6008 | 0.00003 | 2353±3028 | 4342±5811 | <0.00001 |
| **NT-proBNP - month 12 (ng/L)** | 1499±2043 | 3788±5016 | <0.00001 | 1163±1472 | 3414±4316 | <0.00001 |
| **NT-proBNP - relative change (%)** | -18±95 | 19±126 | 0.004 | -28±81 | 20±130 | 0.0001 |

The values are percentage or mean ± standard deviation.

NS = not significant; for other abbreviations see the Table 1
